# Plasma cell mucositis of the larynx

**DOI:** 10.1002/ccr3.1726

**Published:** 2018-07-17

**Authors:** Jonathan G. F. Smith, Caroline P. Smith, Peter J. Leyden

**Affiliations:** ^1^ Craigavon Area Hospital Portadown, Craigavon UK

**Keywords:** airway, mucositis, otolaryngology, steroids, stridor

## Abstract

Stridor is a symptom with a number of causes, usually identified through careful history taking and examination. When the cause is unclear, as in our case, consider investigations such as blood tests, biopsy, and imaging. We discuss a rare diagnosis of plasma cell mucositis, treated with some success by steroids.

## 1 CASE REPORT

A 71‐year‐old male presented to the ENT outpatient clinic with a 2‐year history of mild inspiratory stridor at rest, worsened markedly by exertion but not limiting exercise tolerance. He did not report any voice change, swallowing difficulties, or weight loss. Full examination including flexible nasolaryngoscopy revealed a thickened epiglottis, bulky arytenoids, and aryepiglottic folds only. The patient was a nonsmoker and was taking regular cardiac medications; however, he denied any recent medication changes or new inhalers. There was medical history of cardiac stenting, benign prostatic hypertrophy, and a transient ischemic attack. A first‐degree relative had a previous diagnosis of sarcoidosis.

Initial differential diagnoses included amyloidosis and sarcoidosis, and the patient was commenced on a trial course of oral prednisolone.

Routine bloods were carried out along with immunoglobulins, creatinine kinase, ANCA, ACE, ANA, and serum amyloid A. The results were unremarkable, with the exception of speckled ANA which returned with a positive titer of 40. Following rheumatology review, this was thought to be clinically insignificant.

**Figure 1:**
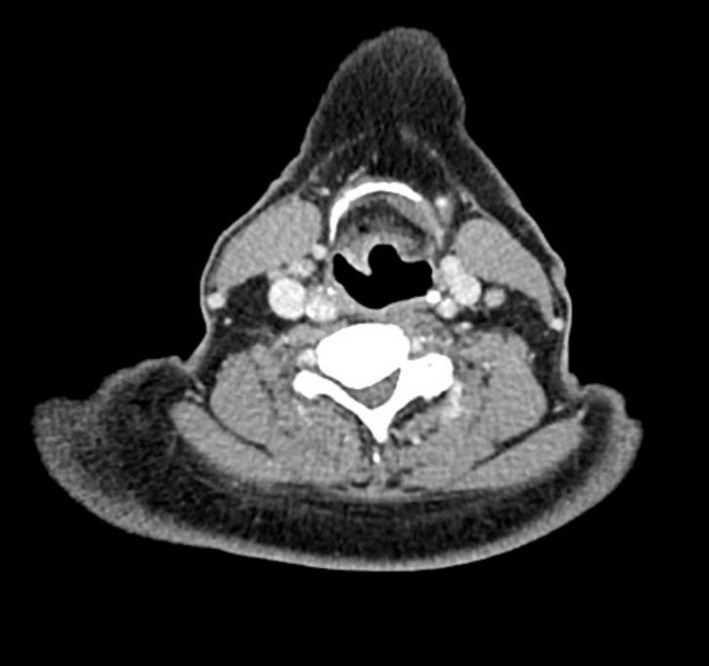
Axial slice of CT of head and neck showing subtle right‐sided thickening of the caudal aspect of the epiglottis

A contrast CT scan of the neck and chest revealed subtle asymmetric thickening of the soft tissue of the epiglottis into the right aryepiglottic fold. There was no lymphadenopathy and no other significant pathology demonstrated. (Figure 1).

The patient was also referred to the respiratory team for an opinion. Pulmonary function tests were essentially normal, with an FEV1 and FVC above 90%.

**Figure 2:**
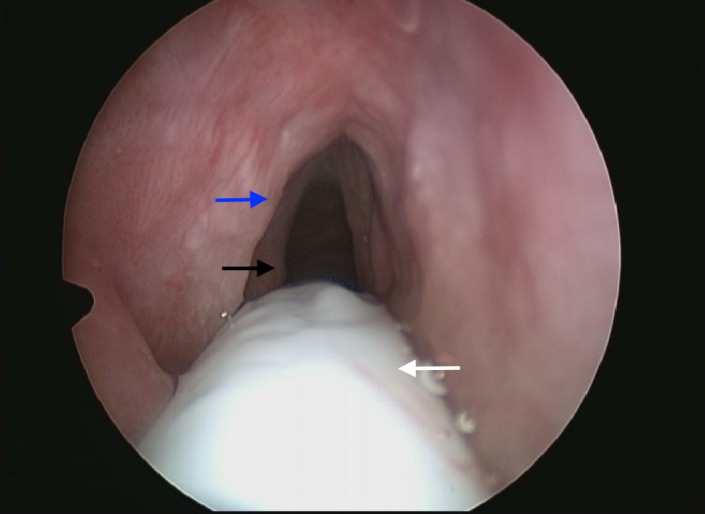
Microlaryngoscopy image showing thickened appearance of supraglottic mucosa. Blue arrow: left false vocal cord. Black arrow: left true vocal cord. White arrow: endotracheal tube

Ongoing laryngeal changes with an increasingly thickened epiglottis were noted at ENT follow‐up, and the patient was booked for microlaryngoscopy and biopsy. This revealed a grossly thickened epiglottis, with marked bilateral vocal cord edema and a generalized cobblestone appearance of the mucosa. There was also evidence of supraglottic narrowing. Biopsies were taken from the epiglottis and supraglottic mucosa. (Figure 2).

**Figure 3:**
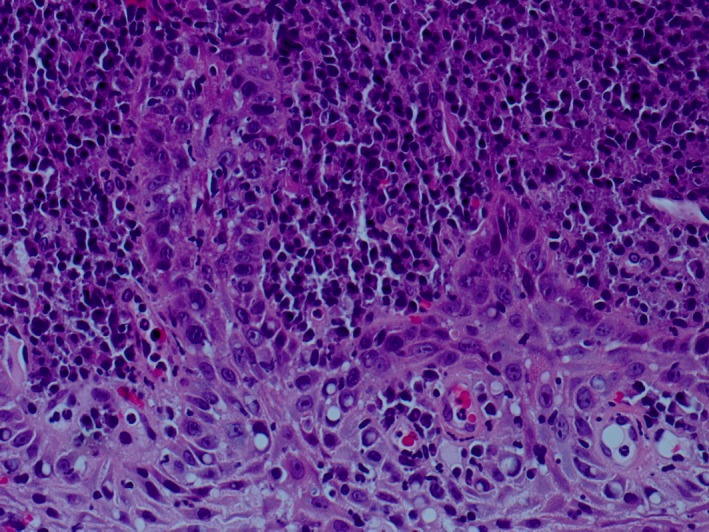
Laryngeal biopsy showing dense infiltration of plasma cells

Histopathological examination revealed moderately hyperplastic stratified squamous epithelium, with elongation of the rete pegs but without cytological atypia. The lamina propria was populated by large numbers of plasma cells in confluent sheets, accompanied by lymphocytes and some neutrophils. The features described, that is, epithelial hyperplasia accompanied by plasmacytosis, were strongly suggestive of a diagnosis of laryngeal plasma cell mucositis. (Figure 3).

The patient was initially commenced on a Pulmicort inhaler; however, this did not lead to any clinical improvement and was subsequently stopped after a number of weeks. He then received a 1‐week course of 40 mg once daily oral prednisolone, followed by a dose decrease of 10 mg every 3 days until stopping completely. This led to partial resolution of symptoms, with a reduction in stridor at rest and on exertion. He is currently not receiving any steroid therapy and is being reviewed on a 6‐monthly basis. This is likely to continue for at least 1 year. The patient has been advised that should his condition deteriorate acutely, a suitable treatment regime would be either a course of oral prednisolone 30 mg or intravenous dexamethasone, depending on the degree of airway compromise.

## 2 DISCUSSION

Plasma cell mucositis (PCM) was first reported as a plasma cell infiltrate of the glans penis by Zoon in 1952.^1^ Similar analogues have been reported in the nose,^2^ lower respiratory tract,^3^ and gingiva, potentially spreading from the latter to the supraglottis.^4^ Isolated plasma cell mucositis of the upper aerodigestive tract is a rarely encountered variant with <50 cases in the literature.^5^ The presence of this pathological process within the larynx was identified in only 10 cases, but these also involved other subsites. There was only one case of isolated laryngeal involvement.^6^

With a macroscopic cobblestone appearance and areas of dense mucosal erythema, PCM is a benign, chronic inflammatory condition of unknown etiology. Numerous reports have made suggestions as to possible causes. A case presented by Tong et al^7^ believed that a toothpaste may be responsible, with the possibility of an ingredient evoking a hypersensitivity reaction. Other case reports have emphasized the presence of severe periodontitis.^5^, ^8^ There is also some suggestion of an association with pre‐existing autoimmune disease,^6^ and this is particularly evident in a case report by Lee et al^9^ discussing a patient with SLE and Sjogren's syndrome.

The differential diagnosis for PCM is wide. Our case highlights that macroscopic appearance may be highly suggestive of amyloidosis or sarcoidosis. A case by Madhavarajan et al^10^ stressed that this pathological phenomenon may also mimic squamous cell carcinoma, and other authors have suggested that pathology such as plasmacytoma and syphilis should be considered.^11^, ^12^ Therefore, early investigation and biopsies are important to target management.

Histologically, our case follows the expected pattern of findings, namely elongated rete pegs, the infrequent presence of both lymphocytes and neutrophils, and a lamina propria populated by large numbers of plasma cells packed in confluent sheets.^13^ Whilst such findings may provide enough evidence for a convincing diagnosis, further techniques may be required to rule out more sinister causes.^14^ Numerous studies have assessed the immunohistochemical profiles of plasma cell infiltrate. Aiba et al^15^ highlighted the predominance of IgG and IgA production, with greater numbers of kappa chains than lambda chains. Cottom et al^16^, whilst defining plasma cell mucositis as a non‐IgG4‐related disease due to lack of histological hallmarks, found that large numbers of IgG4‐positive plasma cells were identifiable in half of their sampled cases. These adjuncts may therefore be useful in offering further confidence in a diagnosis of plasma cell mucositis.

The treatment of PCM is highly contentious with reports noting the use of cryotherapy,^17^ steroid therapy,^15^ and immunosuppressant agents such as tacrolimus,^18^ antibiotics,^19^ or simply removing the source of any potential mucosal irritation^11^ if one is identified. As in our case, a short course of oral corticosteroids appears to be a common initial treatment choice, and whilst it may offer initial symptomatic control, the risks of such therapy must be assessed. Pepper et al^20^ reported a case of squamous cell carcinoma transformation from pre‐existing plasma cell mucositis of the lip and, although the patient in question did not receive oral corticosteroids, postulated that the use of immunosuppressant therapies in this benign condition may have contributed.

A recent case series suggested betamethasone mouthwash and cream may offer some improvement in oral mucositis.^21^ It also reported the novel use of subcutaneous adalimumab with full resolution of symptoms, and this may point to a potential future treatment option.^21^ In any case, the importance of timely and effective medical treatments is highlighted by reports of disease progression requiring surgical intervention. A rare case by Minstry et al discussed the complication of supraglottic stenosis requiring balloon dilatation,^22^ and other cases have noted the requirement of tracheostomy to secure an unsafe airway.^15^ Both radiotherapy^23^ and local debulking^14^ with surgery or carbon dioxide laser have been attempted in the past, and whilst offering an improvement in symptoms, it is thought that it may not halt disease progression.^23^

## 3 CONCLUSION

Plasma cell mucositis is a benign chronic inflammatory condition of unknown etiology. Whilst cases have been reported in the literature affecting the upper aerodigestive tract, our case of isolated laryngeal PCM is rare. Appearances may mimic an autoimmune disorder or malignancy, and therefore, early investigation and biopsy are extremely important to aid diagnosis. Steroids can provide symptomatic relief, and more recently, immune modulators such as adalimumab have been reported as beneficial. We suggest regular clinical follow‐up in such cases to assess for disease progression and to allow intervention as required.

## CONFLICT OF INTEREST

None declared.

## AUTHORSHIP

JS: contributed with literature review, collation of literature findings, and drafting of the manuscript and discussion. CS: helped to write the article, with grammatical and syntax review and revision of the manuscript and image captions. PL: contributed with retrieval and selection of images, and critical review and revision of the manuscript.
